# Editorial

**Published:** 1839-01-26

**Authors:** 


### THE MEDICAL EXAMINER.

PHILADELPHIA, JAN. 26, 1839.

We commence, in the present number, the publication of a series of reports from the Philadelphia Hospital, Blockley. The first article is a report of the Men's Lunatic Asylum. The subject of the management of the insane, and of the erection of suitable hospitals for their reception, is not a mere question of local interest, but will occupy a future occasion.

